# Megakaryocytic Leukemia 1 (MKL1) Regulates Hypoxia Induced Pulmonary Hypertension in Rats

**DOI:** 10.1371/journal.pone.0083895

**Published:** 2014-03-19

**Authors:** Zhibin Yuan, Jian Chen, Dewei Chen, Gang Xu, Minjie Xia, Yong Xu, Yuqi Gao

**Affiliations:** 1 Department of Pathophysiology and High Altitude Physiology, College of High Altitude Military Medicine, Third Military Medical University, Chongqing, China; 2 Key Laboratory of High Altitude Medicine, Ministry of Education, Third Military Medical University, Chongqing, China; 3 Key Laboratory of High Altitude Medicine, PLA, Third Military Medical University, Chongqing, China; 4 Key Laboratory of Cardiovascular Disease, Department of Pathophysiology, Nanjing Medical University, Nanjing, Jiangsu, China; University of Giessen Lung Center, Germany

## Abstract

Hypoxia induced pulmonary hypertension (HPH) represents a complex pathology that involves active vascular remodeling, loss of vascular tone, enhanced pulmonary inflammation, and increased deposition of extracellular matrix proteins. Megakaryocytic leukemia 1 (MKL1) is a transcriptional regulator known to influence cellular response to stress signals in the vasculature. We report here that in response to chronic hypobaric hypoxia, MKL1 expression was up-regulated in the lungs in rats. Short hairpin RNA (shRNA) mediated depletion of MKL1 significantly ameliorated the elevation of pulmonary arterial pressure *in vivo* with a marked alleviation of vascular remodeling. MKL1 silencing also restored the expression of NO, a key vasoactive molecule necessary for the maintenance of vascular tone. In addition, hypoxia induced pulmonary inflammation was dampened in the absence of MKL1 as evidenced by normalized levels of pro-inflammatory cytokines and chemokines as well as reduced infiltration of pro-inflammatory immune cells in the lungs. Of note, MKL1 knockdown attenuated fibrogenesis in the lungs as indicated by picrosirius red staining. Finally, we demonstrate that MKL1 mediated transcriptional activation of type I collagen genes in smooth muscle cells under hypoxic conditions. In conclusion, we data highlight a previously unidentified role for MKL1 in the pathogenesis of HPH and as such lay down groundwork for future investigation and drug development.

## Introduction

Hypoxia induced pulmonary hypertension (HPH) is a debilitating disease that will eventually lead to right ventricular failure [1]. HPH represents a complicated pathophysiological process that includes a series of interconnected events. Although the precise mechanism underlying the pathogenesis of HPH is largely unknown, it is generally believed that active vascular remodeling as a result of smooth muscle cell proliferation, increased pulmonary inflammation due to leukocyte adhesion and aggregation, disruption of vascular tone, and accelerated fibrogenesis all play a critical role. Importantly, the gene expression profile within the lungs is altered significantly in response to hypoxic stress [2]. For instance, it has been documented that accompanying pulmonary inflammatory response, the production and release of a number of cytokines, including IL-6 and TNF-α, are markedly up-regulated [3], [4]. Another exemplary alteration of gene expression taking place in the lungs is the induction of extracellular matrix (ECM) proteins such as type I collagen in smooth muscle cells [5], [6]. How these diverse transcriptional events are coordinated remains obscure.

Megakaryocytic leukemia 1 (MKL1), also termed myocardin-related transcription factor A (MRTF-A), belongs a family of transcriptional regulators initially reported to be involved in the phenotypic modulation of smooth muscle cells [7], [8]. Several recent investigations have strongly indicated that MKL1 may function as a stress protein orchestrating cellular response to a range of extrinsic and intrinsic insults. It has been demonstrated the MKL1 participates in ischemia induced cardiac remodeling by regulating type I collagen transcription in fibroblast cells [9]. Meanwhile, MKL1 has shown to mediate the hypertrophic response in mice by activating the transcription of brain natriuretic peptide (BNP) gene [10]. Recently, Fang et al have reported that MKL1 mediates the deleterious effects of oxLDL, a major risk factor for atherosclerosis, by up-regulating intercellular adhesion molecule 1 (ICAM-1) transcription while simultaneously down-regulating NO synthase (eNOS) transcription in vascular endothelial cells [11]. In light of these findings, we hypothesized that MKL1 might be a key player in the pathogenesis of HPH. Our data as presented here suggest that MKL1 expression is elevated in the lungs in rats with HPH and that MKL1 silencing ameliorates HPH. Therefore, targeting MKL1 may yield novel therapeutic solutions for the intervention of HPH in the future.

## Materials and Methods

### Cell culture, plasmids, and transfection

Rat vascular smooth muscle cells (A10) were cultured in DMEM as described previously [12]. Primary human pulmonary arterial smooth muscle cells (Lonza) were maintained SMBM supplemented with growth factors supplied by the vendor. Where indicated, hypoxia (1% O_2_) was achieved by a mixture of ultra-high purity gases (5% CO_2_, 10% H_2_, 85% N_2_) in a 37°C incubator (Thermo Fisher). MKL1 expression construct, shRNA plasmid targeting MKL1, *col1a2* promoter luciferase construct, and *col1a1* promoter luciferase construct have been described previously [11], [13], [14]. Small interfering RNA sequences for rat MKL1 were as follows: #1, CAGGUGAAUUACCCAAAGGUATT, and #2, UGGAGCUGGUGGAGAAGAATT. Transient transfections were performed with Lipofectamine 2000 (Invitrogen). Experiments were routinely performed in triplicate wells and repeated three times.

### Animals and *in vivo* gene silencing

All animal experiment protocols were approved by the Committee on Ethical Practice of Animal Studies of the Third Military Medical University. Briefly, 8-week old male Sprague-Dawley rats were housed in a closed chamber with an ambient air pressure of 405.35 mmHg (approx. 0.53atm, or equivalent of 5000 m altitude, or equivalent of 11.2% O_2_) for 4 weeks to induce pulmonary hypertension. shRNA targeting MKL1 (CATGGAGCTGGTGGAGAAGAA) was cloned into a SuperSilencing lentiviral vector. At week 1 and week 3, these rodents were injected via sublingual vein purified lentivirus (1×10^8^ viral particles/per injection). Detailed description for the measurement of hemodynamic parameters Key cardiac/pulmonary metrics can be found in the supplementary material.

### Isolation of pulmonary arteries from SD rats

Isolation of pulmonary arteries was performed essentially as described before [15]. Briefly, the rats were anesthetized with amobarbital. The lungs were removed from the chest cavity and rinsed with washing buffer (138 mM NaCl, 4.7 mM KCl, 1.2 mM NH2PO4, 1.2 mM MgSO4, 1.8 mM CaCl2, 5 mM HEPES and 10 mM glucose). The superficial tissue and the bronchus artery were discarded with fine micro-scissors. The adventitia is carefully removed from isolated arteries under a dissection scope. Tertiary lobular branches (≤300 µm) were used for subsequent experiments.

### Measurement of hemodynamics

Following the development of HPH, rats were anesthetized and the right internal jugular vein was dissected. A pulmonary artery catheter (PV 1, 0.28 mm diameter) was inserted through an introducer under pressure waveform monitoring and pulmonary arterial pressure was recorded. A PE-50 catheter was inserted into the left carotid artery with connection to a digital blood pressure analyzer (BPA, Micro-Med. Inc., Louisville, KY) for continuous recordings of systolic, diastolic and mean arterial blood pressures and heart rate.

### Morphometric analysis

Wall thickness was measured with an ocular micrometer and expressed as the medial wall thickness (the distance between the internal and external lamina) divided by the diameter of the vessel (the distance between the external lamina). Muscularity was determined using α-SMA as a marker. Each vessel was categorized as fully muscular (>75% α-SMA staining), partially muscular (<75% α-SMA staining), or nonmuscular (no α-SMA staining), and values were expressed as the percentage of total vessels. Percent medial thickness was determined using Image J as previously described [16]. For each animal, at least 20 different fields and 100 different vessels (50–200 µM) were scored.

### Protein extraction and Western blotting

For cells, lysates were obtained by re-suspending cell pellets in RIPA buffer (50 mM Tris pH 7.4, 150 mM NaCl, 1% Triton X-100) with freshly added protease inhibitor tablet (Roche). For tissues, lysates were obtained by homogenizing samples in lysis buffer (10 mM Tris pH 8.0, 130 mM NaCl, 1% Triton-X100). Western blot analyses were performed with anti-β-actin (Sigma; 1∶5,000), anti-collagen type I (Rockland; 1∶5,000), and anti-MKL1 (Santa Cruz; 1∶1,000) antibodies.

### Enzyme-linked immune absorbance assay (ELISA)

ELISA was performed using rat pulmonary artery homogenates to measure MCP-1/CCL2 (Pierce), RANTES/CCL5 (Invitrogen), IL-6, TNF-α, MIP-1/CCL4 (USCN Life Sciences), and TGF-β (R&D) as described previously [17].

### RNA Isolation and Real-time PCR

RNA was extracted with the RNeasy RNA isolation kit (Qiagen) as described before [18], [19]. Reverse transcriptase reactions were performed using a SuperScript First-strand Synthesis System (Invitrogen). Real-time PCR reactions were performed on an ABI Prism 7500 system. Primers are listed in Table S1.

### Histology

Immunohistochemistry was performed as previously described [20]. Briefly, the sections were blocked with 10% normal goat serum for 1 hour at room temperature and then incubated with anti-MKL1 (Santa Cruz; 1∶100), anti-sm-MHC (Sigma; 1∶100), anti-α-SMA (Sigma; 1∶100), anti-CD3 (BD Bioscience; 1∶100), anti-CD45 (Abcam; 1∶100), or anti-F4/80 (Abcam; 1∶100) antibodies. Staining was visualized by incubation with an appropriate biotinylated 2° antibody and developed with a streptavidin-horseradish peroxidase kit (Pierce) for 20 min. Sections were counterstained with hematoxylin. Pictures were taken using an Olympus IX-70 microscope. Picrosirius red, Masson's trichrome (both from Sigma), and elastica von Gieson (Millipore) stainings were performed according to vendor's recommendations.

### Immunofluorescence staining

The plastic-embedded sections were incubated with primary antibodies, anti-α-SMA and anti-Von Willebrand factor (both from Abcam), followed by incubation with rabbit secondary antibodies (Jackson ImmunoResearch). The nuclei were counterstained with DAPI (Sigma).

### NO measurement

NO measurement has been described before [11]. Briefly, tissues were pre-heated to 95°C for 5 min to denature proteins and then homogenized in Kreb's solution (118 mM NaCl, 4.6 mM KCl, 27.2 mM NaHCO3, 1.2 mM MgSO4, 2.5 mM CaCl2, 1.2 mM KH2PO4, and 11.1 mM glucose) for 1 hour at 37°C. Afterwards, 100 µl supernatant was collected and the nitrate content was measured with a Griess reagent system (Promega).

### Statistical Analysis

One-way ANOVA with post-hoc Scheffe analyses were performed using an SPSS package. p values smaller than .05 were considered statistically significant and designated *.

## Results

### MKL1 expression is up-regulated in pulmonary arteries by hypoxia in rats

**Figure 1:**
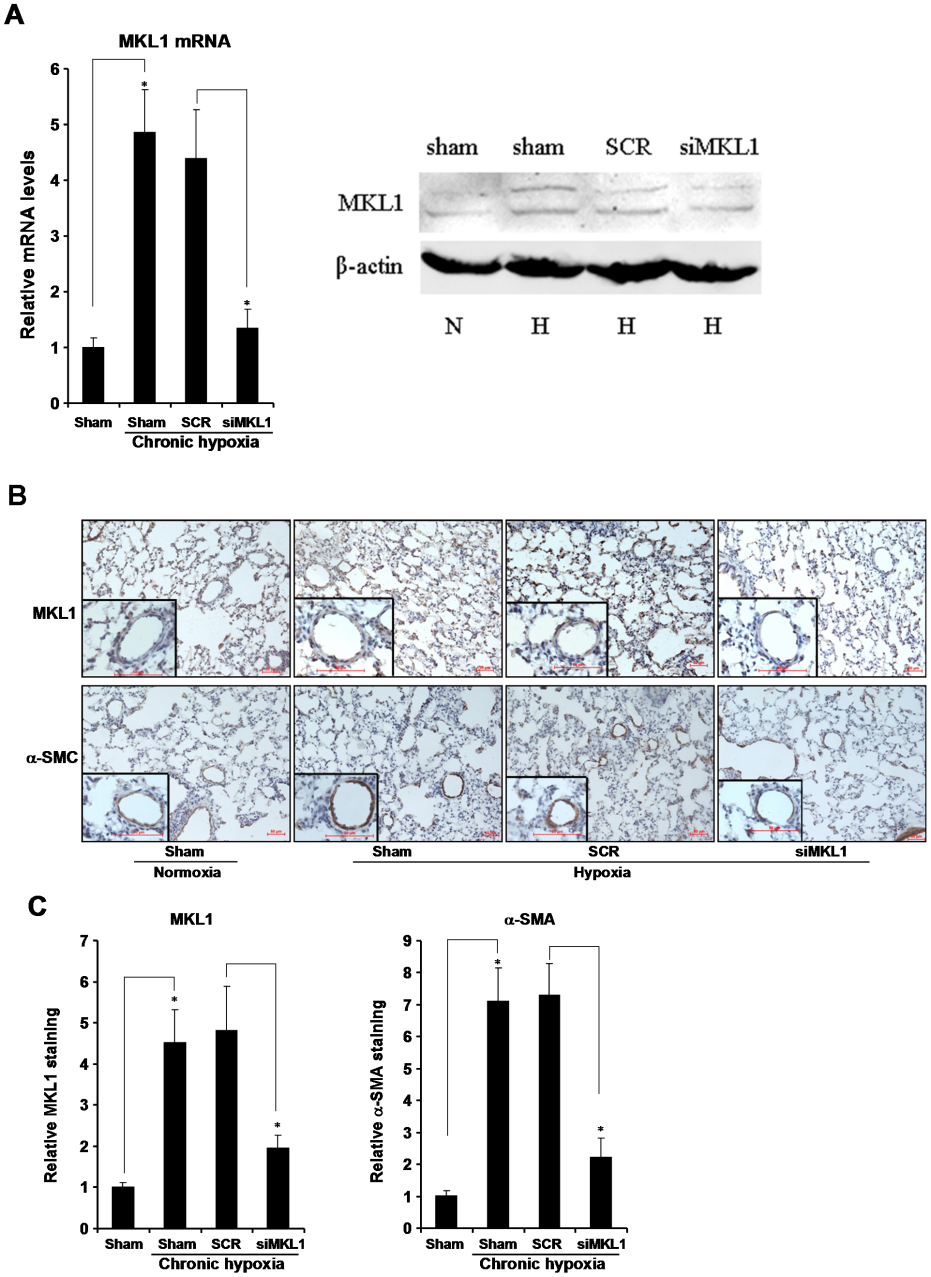
MKL1 expression is up-regulated in the lungs in rats with hypoxia-induced pulmonary hypertension. Sprague Dawley rats were injected with lentiviral particles carrying shRNA targeting MKL1 or random shRNA (SCR) and induced to develop HPH as described under *Methods*. (A) MKL1 mRNA and protein levels in pulmonary arteries were assessed by qPCR and Western blotting. (**B**) MKL1 and α-SMA levels in pulmonary arteries were examined by immunohistochemistry. (**C**) Protein expression of MKL1 and α-SMA was quantified by Image Pro and expressed as relative staining compared to the control group set as 1. N = 10 rats for each group.

We first evaluated the expression levels of MKL1 in the lungs of rats that had been allowed to develop HPH when housed in a hypoxic chamber for 4 weeks. Quantitative PCR (qPCR) assays revealed that mRNA level of MKL1 was significantly (by 486%) induced in pulmonary arteries rats with HPH as opposed to control rats; MKL1 protein level as examined by Western blotting was also increased in hypoxic rats (Fig. 1A). By comparison, MKL1 mRNA expression was only modestly up-regulated (by 203%) in aortic arteries (Fig. S1A). These data were corroborated by immunohistochemistry staining, which demonstrated that MKL1 was strongly stimulated in pulmonary arteries in rats in response to chronic hypoxia (Fig. 1B, 1C, S1B); there was a partial overlapping of MKL1 expression and α-SMA expression, indicating the presence of MKL1 in the vessel wall. We also probed the expression of MKL1 in cultured rat vascular smooth muscle cells (A10) and human primary pulmonary arterial smooth muscle cells (HPASMC) challenged with 1% O_2_. Both mRNA and protein levels of MKL1 were increased by low oxygen tension (Fig. S1C, S1D). Together, our results indicate that MKL1 is activated in the lungs in response to hypoxic challenge *in vivo and in vitro*.

### MKL1 silencing attenuates hypoxia-induced pulmonary hypertension in rats

Next, we assessed the possibility that MKL1 silencing might avert HPH in rats. To this end, we injected lentivirus carrying either shRNA targeting MKL1 (siMKL1) or random shRNA (SCR) into rats via the sublingual vein. As a result, MKL1 expression was suppressed in pulmonary arteries (Fig. 1A, 1B), but not in aortic arteries (Fig. S1A, S1B), at both mRNA and protein levels.

**Figure 2:**
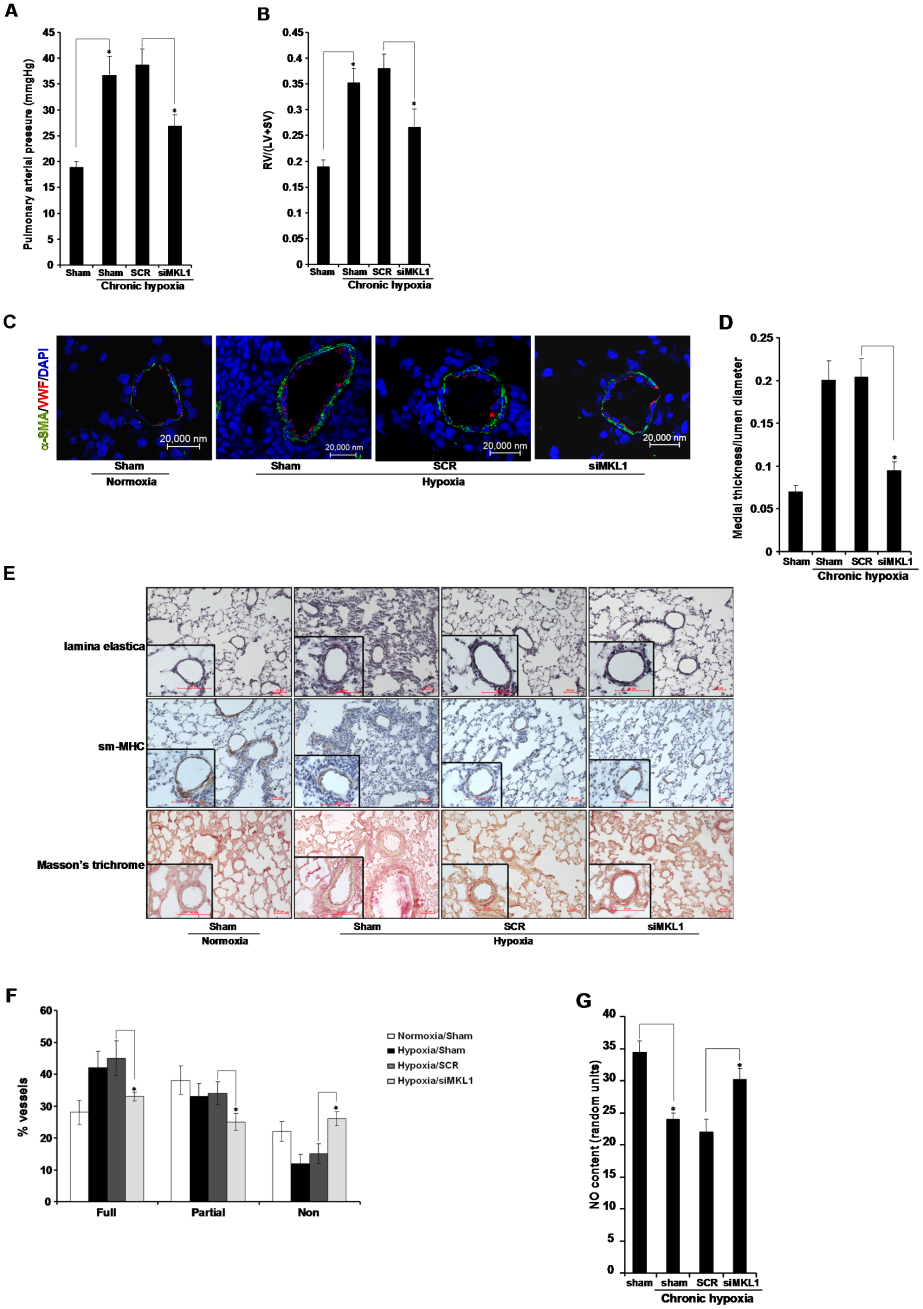
MKL1 silencing attenuates hypoxia-induced pulmonary hypertension in rats. Sprague Dawley rats were injected with lentiviral particles carrying shRNA targeting MKL1 or random shRNA (SCR) and induced to develop HPH as described under *Methods*. (**A**) Pulmonary arterial pressure was measured. N = 10 rats for each group (**B**) RV/(LV+SV) was measured to assess right ventricle hypertrophy. N = 10 rats for each group (**C, D**) Immunofluorescence staining was performed with anti-α-SMA (orange) and anti-VWF (green). Medial thickness and lumen diameter were measured by Image J. N = 10 rats for each group (**E, F**) Histochemical stainings were performed as described under Methods. Muscularization of pulmonary vessels was evaluated as described in Methods. N = 10 rats for each group (**G**) Levels of NO were evaluated by a commercially available kit as described under *Methods*. N = 5 rats for each group.

Depletion of MKL1 by shRNA resulted in a marked reduction of pulmonary arterial pressure (Fig. 2A) and significantly attenuated right ventricular hypertrophy (Fig. 2B), indicating that MKL1 indeed is required for the development of HPH *in vivo*. Meanwhile, neither systemic blood pressure nor heart rate was impacted by MKL1 knockdown (Fig. S2A, S2B). Of note, MKL1 silencing in rats under normoxic conditions did not alter pulmonary arterial pressure, or systemic blood pressure, or heart rate (Fig. S2C–S2E), suggesting that MKL1 is dispensable for maintaining cardiopulmonary function under physiological conditions. Expansion of the pulmonary vessel wall marks a critical step in the pathogenesis of HPH [21]. MKL1 silencing blocked this active vascular remodeling as measured by the thickness of the medial layer (Fig. 2C, 2D). MKL1 knockdown also alleviated muscularization of pulmonary vessels (Fig. 2E, 2F). Endothelium-derived NO serves as a key vasodilator that helps maintain the vascular tone [22]. As expected, NO levels were decreased in the lungs in HPH rats, but were normalized in the absence of MKL1 (Fig. 2G). Combined, these results suggest that MKL1 might be a critical regulator of HPH *in vivo* by influencing vascular remodeling and vascular tone.

### MKL1 silencing attenuates hypoxia-induced pulmonary inflammation in rats

**Figure 3:**
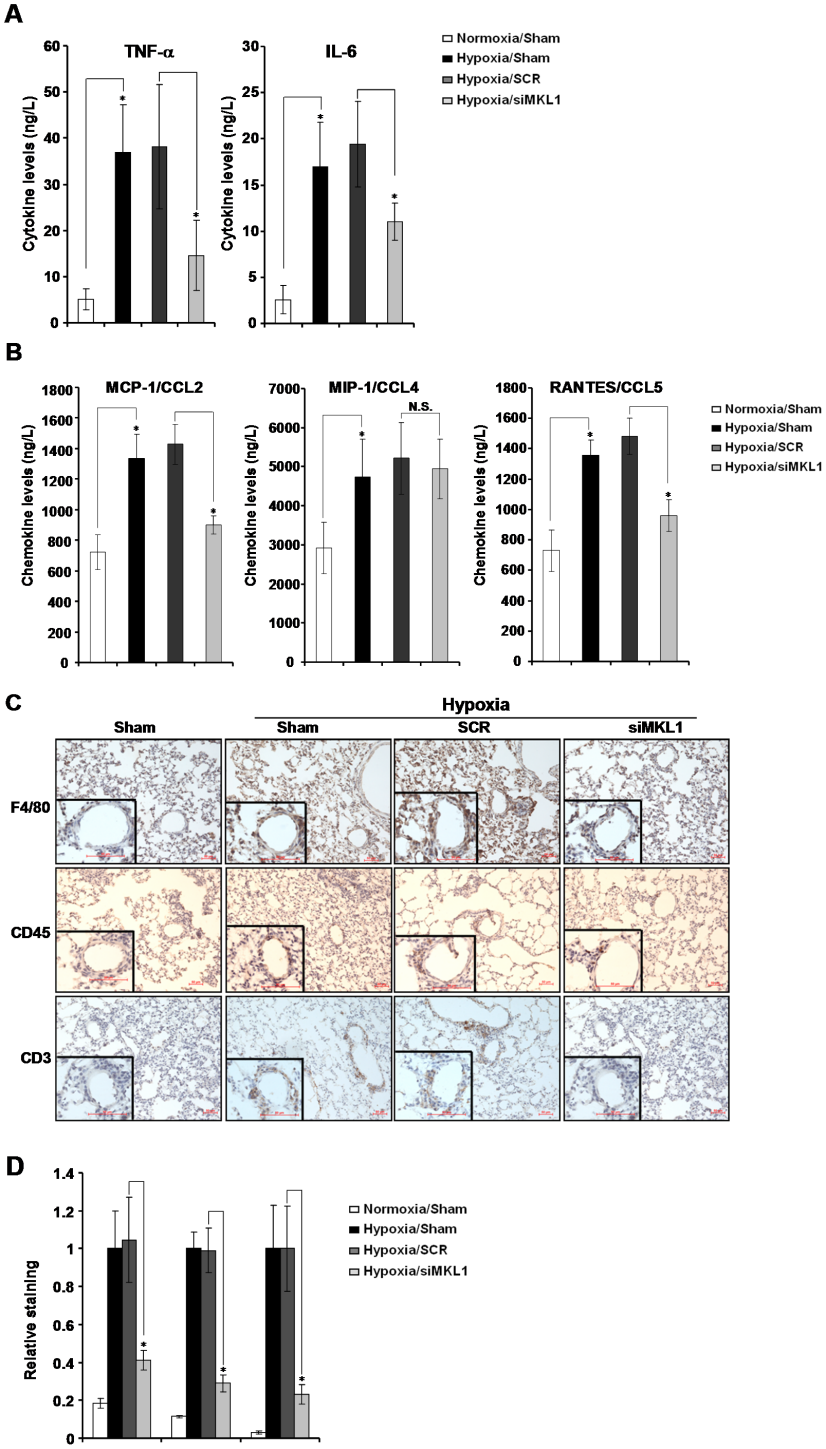
MKL1 silencing attenuates hypoxia-induced pulmonary inflammation in rats. Sprague Dawley rats were injected with lentiviral particles carrying shRNA targeting MKL1 or random shRNA (SCR) and induced to develop HPH as described under *Methods*. (**A, B**) Levels of cytokines and chemokines were assessed by ELISA. N = 5 rats for each group (**C, D**) Immunohistochemistry was performed with indicated antibodies as described under *Methods* and quantified by Image J. N = 5 rats for each group.

In the lungs challenged with hypoxia, there is an increased adhesion and aggregation of immune cells creating a pro-inflammatory milieu [23], [24]. These immune cells, in turn, may secrete inflammatory mediators to promote the pathogenesis of HPH [25], [26]. Indeed, production of both TNF-α and IL-6 were both increased in the lungs in HPH rats (Fig. 3A). MKL1 elimination, however, potently suppressed the synthesis of these cytokines. Chemokines, such as MCP-1/CCL2, MIP-1/CCL4, and RANTES/CCL5, are responsible for the recruitment of immune cells to the lung to initiate pro-inflammatory response [27]. As expected, all three chemokines were up-regulated by hypoxia in rats (Fig. 3B). On the other hand, MKL1 knockdown was able to neutralize the induction of CCL2 and CCL5, but no CCL4. We then directly assessed the effect of MKL1 silencing on the recruitment of immune cells to the lungs by immunohistochemistry. As shown in Fig. 3C, chronic hypoxia resulted in a significant increase in the number of macrophages (labeled by F4/80), leukocyte (labeled by CD45), and T lymphocyte (labeled by CD3) within the lungs. MKL1 loss-of-function abrogated the adhesion and aggregation of all three types of immune cells (Fig. 3D). Collectively, these results suggest that MKL1 may play a role in establishing and/or maintaining the pro-inflammatory microenvironment in the lungs in HPH rats.

### MKL1 silencing attenuates hypoxia-induced pulmonary fibrogenesis in rats

**Figure 4:**
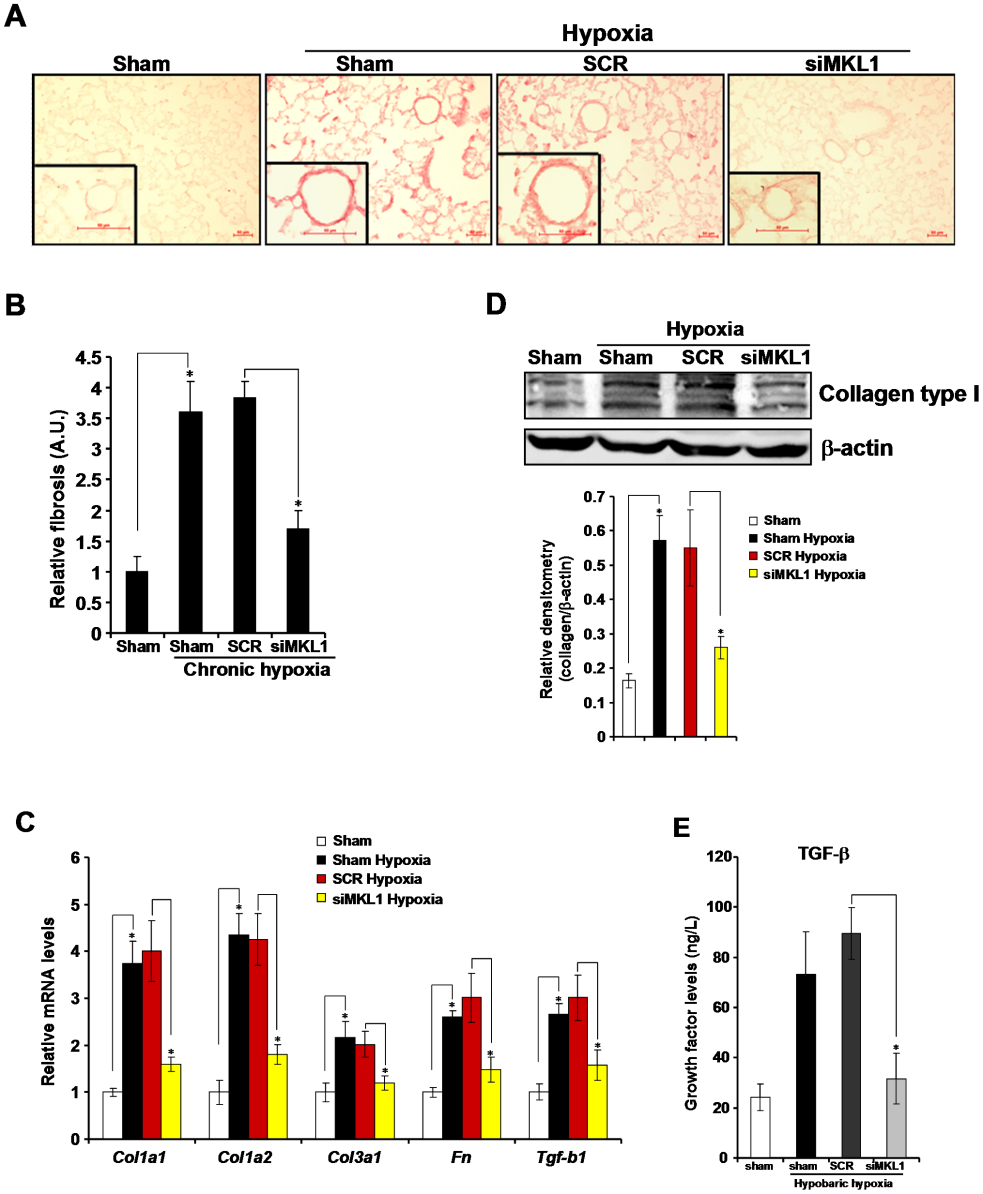
MKL1 silencing attenuates hypoxia-induced pulmonary fibrogenesis in rats. Sprague Dawley rats were injected with lentiviral particles carrying shRNA targeting MKL1 or random shRNA (SCR) and induced to develop HPH as described under *Methods*. (**A**) Picrosirius red staining was performed as described under *Methods*. (**B**) Positive staining was quantified by Image J and expressed as relative fibrosis compared to the control group. A. U., arbitrary unit (**C, D**) Expression of molecules involved in fibrogenesis in pulmonary arteries was measured by qPCR (C) and Western blotting (D). N = 5 rats for each group (**E**) Levels of TGF-β in pulmonary arteries were assessed by ELISA. N = 4 rats for each group.

At late stages of HPH, there is an increase in the production of extracellular matrix (ECM) proteins, collagen type I being the most prominent one, in the lungs leading to pulmonary fibrosis [28]. We first examined whether MKL1 could alter collagen deposition in the lungs in HPH rats. As shown in Fig. 4A and Fig. 4B, more collagen fibers were present in the lungs of HPH rats whereas MKL1 deletion caused a significant reduction of collagen secretion. By using qPCR, we confirmed that induction of a panel of fibrogenic genes under hypoxic conditions, including type I collagen (col1a1, col1a2), type III collagen (col1a3), fibronectin (fn) and transforming growth factor (tgf-b1), was all down-regulated in the absence of MKL1 in pulmonary arteries (Fig. 4C). MKL1 silencing also led to a decrease in protein expression of type I collagen (Fig. 4D). In accordance, MKL1 depletion prevented the accumulation of TGF-β proteins in the lungs (Fig. 4E).

**Figure 5:**
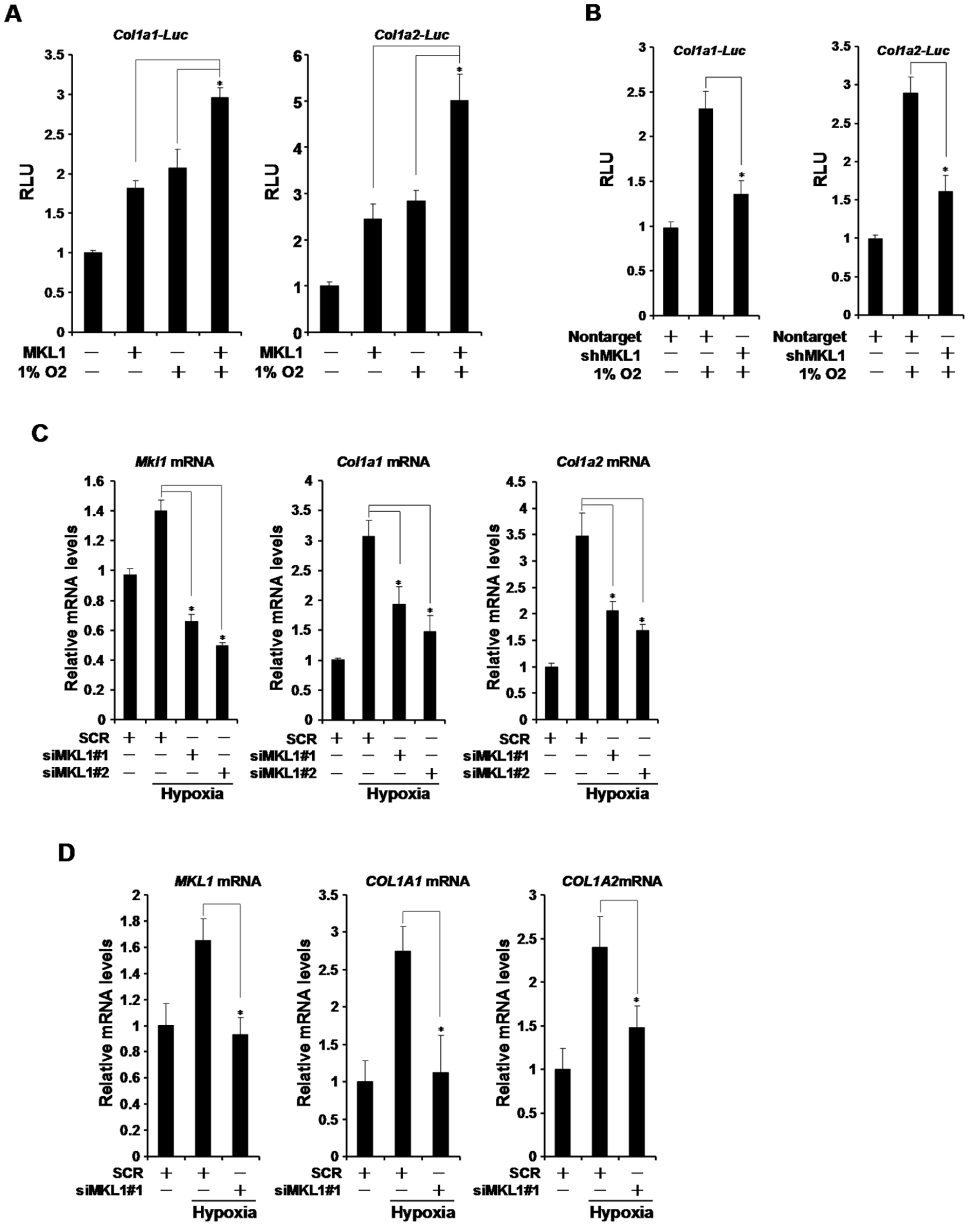
MKL1 regulates hypoxia-induced collagen transcription in smooth muscle cells. (**A**) Col1a1 or col1a2 promoter luciferase construct was transfected into A10 cells with MKL1 expression construct followed by exposure to 1% O2 for 24 hours. Luciferase activities were expressed as relative luciferase unit (RLU). (**B**) Col1a1 or col1a2 promoter luciferase construct was transfected into A10 cells with shRNA plasmid targeting MKL1 or a control shRNA plasmid (nontarget) followed by exposure to 1% O2 for 24 hours. Luciferase activities were expressed as RLU. (**C, D**) A10 (C) or primary human pulmonary arterial smooth muscle (D) cells were transfected with MKL1 specific or control siRNA (SCR) followed by exposure to 1% O2 for 24 hours. Expression of collagen type I genes was measured by qPCR.

Vascular smooth muscle cells (VSMC) are one of the major sources of ECM production in the lungs [5], [6]. To further verify the role of MKL1 in hypoxia-induced collagen production, we transfected collagen type I gene promoter (col1a1 or col1a2) luciferase construct into cultured rat VSMC (A10). Hypoxia activated the transcription of type I collagen genes (Fig. 5A). Over-expression of MKL1 further potentiated the transcriptional activation of type I collagen genes. In contrast, knockdown of MKL1 by shRNA abolished the induction of collagen transcription by hypoxia (Fig. 5B). Finally, small interfering RNA (siRNA) mediated depletion of MKL1 prevented the increased synthesis of endogenous collagen type I mRNA in VSMC under hypoxic conditions (Fig. 5C, 5D). Taken together, MKL1 may participate in hypoxia-induced fibrogenesis in the lungs by transcriptionally activating collagen type I genes.

## Discussion

Hypoxic pulmonary hypertension (HPH) is a devastating disease that eventually leads to right heart failure and death. Although there is a lack of unifying model for the pathogenesis of HPH, it is generally agreed that accumulation of pro-inflammatory mediators in the lungs and vascular remodeling as a result of extracellular matrix over-production likely provide two of the most important links [29]. Here we report that shRNA mediated silencing of MKL1, a multifaceted transcriptional modulator, effectively ameliorated HPH in rats by dampening pulmonary inflammation and normalizing collagen type I synthesis in smooth muscle cells.

Increased production and release of pro-inflammatory mediators have been observed in the plasma of patients with HPH [3], [30], [31]. Accumulation of pro-inflammatory mediators will injure vascular endothelium, promote the encroachment of medial smooth muscle cells to actively remodel pulmonary vasculature, and encourage the adhesion and aggregation of immune cells to the vessel wall, all of which cause irreparable damages and exacerbate HPH. At the transcriptional level, expression of these cytokines and chemokines depend on NF-κB, the master regulator of the innate immunity [32]. Indeed, it is well documented that hypoxia activates NF-κB to initiate and perpetuate a pro-inflammatory microenvironment in the context of pulmonary hypertension [33]–[35]. In the present study, we have found that when MKL1 was depleted by shRNA, levels of pro-inflammatory mediators were significantly down-regulated in the lungs of rats with HPH (Fig. 3A, 3B). In addition, recruitment of immune cells was greatly diminished (Fig. 3C, 3D). Our previous findings suggest that MKL1 directly interacts with NF-κB and potentiates NF-κB dependent transcription [11]. Therefore, it is possible that MKL1 may influence the synthesis of these cytokines and chemokines in a NF-κB dependent manner in immune cells. On the other hand, the interaction between immune cells and vascular endothelial cells that serves as a prerequisite for extravasation relies on the expression of a group of cell-cell adhesion molecules such as ICAM-1 and VCAM-1 [36], [37]. MKL1 has been shown to promote leukocyte adhesion by inducing ICAM-1 and VCAM-1 transcription in endothelial cells [11]. Thus, an equally plausible explanation for decreased infiltration of immune cells in the lungs following MKL1 knockdown would be that endothelial cells cannot produce sufficient amount of adhesion molecules to attract and sustain the interaction with immune cells. Future investigations employing tissue-specific MKL1 knockout animal models will be able to differentiate these two possibilities.

Another important finding presented here is that MKL1 silencing attenuated pulmonary fibrosis in rats with HPH (Fig. 4). In response to hypoxia, pulmonary vascular smooth muscle cells undergo several changes typical to HPH, including accelerated proliferation and migration and augmented ability to synthesize ECM proteins such collagen and fibronectin [38]. Collagen type I is the most prominent component of ECM in the lungs and pulmonary fibrosis as a result of enhanced collagen transcription is deemed a hallmark event in HPH [39]. We show here that MKL1 is both sufficient and necessary for hypoxia-induced collagen type I transactivation in smooth muscle cells (Fig. 5). Recently, two research groups have identified collagen type I as a direct transcriptional target for MKL1. Small et al propose that MKL1 is recruited to the collagen promoter by serum response factor (SRF) in cardiac fibroblast challenged with ischemia [9]. Luchsinger et al, in the meantime, suggest that Sp1 is responsible for bringing MKL1 to the collagen promoter to activate transcription in lung fibroblast [40]. Both SRF and Sp1 can be activated by hypoxia themselves and are known to mediate a range of cellular responses to hypoxia [41]–[43]. In light of our observation that MKL1 was up-regulated by hypoxia in the lungs (Fig. 1A, 1B), it is conceivable that a large transcriptional complex containing MKL1, SRF, and/or Sp1 could be assembled on the collagen promoter in response to hypoxia in smooth muscle cells. Alternatively, we have also observed that induction of TGF-β, a major pro-fibrogenic growth factor, was blunted in the absence of MKL1, suggesting that TGF-β might be a direct transcriptional target of MKL1. Of note, Parmacek and colleagues have recently discovered that MKL2, a closely related family member of MKL1, directly activates TGF-β transcription during vascular development [44]. Since TGF-β is responsible for the synthetic ability of smooth muscle cells, we propose that MKL1 may exert its pro-fibrogenic effect, at least in part, through activating TGF-β expression in the lungs. Still, another possibility is that the observed improvements of pulmonary function were a consequence of MKL1 blocking in the heart since Small et al have shown that MKL1 deficiency alleviates cardiac infarction [9]. In essence, systemic MKL1 expression on hemodynamics under chronic hypoxia cannot be excluded at this point. Tissue-specific deletion of MKL1 will likely shed more light on dissolving this issue in the future.

In conclusion, our data have suggested a potential role for MKL1 in the pathogenesis of HPH. In order for MKL1 to be targeted in the prevention and/or treatment of HPH, future research should scrutinize the role of MKL1 in more relevant animal models (e.g., Sugen-induced HPH) and probe the tissue-specific role of MKL1 in HPH.

## Supporting Information

Figure S1 (**A, B**) Sprague Dawley rats were injected with lentiviral particles carrying shRNA targeting MKL1 or random shRNA (SCR) and induced to develop HPH as described under *Methods*. MKL1 mRNA (A) and protein (B) levels in aortic arteries were assessed by qPCR and immunohistochemistry. N = 5 mice for each group (**C, D**) A10 cells (C) and HPASMCs (D) were exposed to 1% O_2_ and harvested at indicated time points. mRNA and protein levels of MKL1 were measured by qPCR and Western.
(PDF)

Figure S2 (**A, B**) Sprague Dawley rats were injected with lentiviral particles carrying shRNA targeting MKL1 or random shRNA (SCR) and induced to develop HPH as described under *Methods*. Systemic blood pressure (A) and heart rate (B) were measured by using the PowerLab data acquisition system. N = 5 mice for each group (**C–E**) Sprague Dawley rats were injected with lentiviral particles carrying siMKL1 or SCR and maintained under normoxic conditions for 4 weeks. Pulmonary arterial pressure (C), systemic blood pressure (D), and heart rate (E) were recorded. N = 5 mice for each group
(PDF)

Table S1 Real-time quantitative PCR primers.
(PDF)
