# Correction: Megakaryocytic Leukemia 1 (MKL1) Regulates Hypoxia Induced Pulmonary Hypertension in Rats

**DOI:** 10.1371/journal.pone.0350157

**Published:** 2026-05-27

**Authors:** Zhibin Yuan, Jian Chen, Dewei Chen, Gang Xu, Minjie Xia, Yong Xu, Yuqi Gao

Following publication of this article [1], the following errors were identified in Fig 3:

- There is an error in the group sample size reported for Hypoxia/Sham and Hypoxia/SCR groups in the legend of Fig 3A-B of [1]. Please see the complete and correct Fig 3 provided with this Correction.
- In Fig 3C, the CD3 siMKL1 panel is incorrect and is a duplicate of the CD3 Sham panel.
- In Fig 3D, the X axis labels are missing from the bar chart and there are errors in the quantitative data reported in the chart.

With this Correction, the authors provide a revised Fig 3 in which the incorrect panel in Fig 3C is replaced with the correct image from the original experiments and the Fig 3D bar chart is revised to address both the missing axis labels and errors in the originally published Fig 3D. The authors confirm that these changes do not affect the results and conclusions.

The authors provide additional methodological information as follows:

Animal health and behavior were closely monitored by the researchers on a daily basis. Specifically, both general signs and respiration were examined to ensure that the rats did not display any of the following abnormalities: significant weight loss, anorexia, dehydration, difficulty of mobility, lethargy, bleeding, hypothermia, dyspnea, and gasping. The rats were euthanized by CO_2_ inhalation (30% chamber volume displacement per minute).

With this Correction, the corresponding author shares the available image and quantitative data underlying the corrected Fig 3 (S1-2 Files). They confirm that the original data underlying all other figures are available.

**Fig 3:**
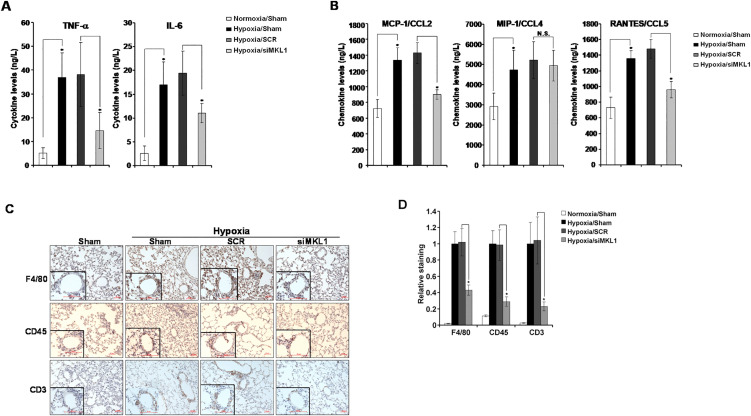
MKL1 silencing attenuates hypoxia-induced pulmonary inflammation in rats. Sprague Dawley rats were injected with lentiviral particles carrying shRNA targeting MKL1 or random shRNA (SCR) and induced to develop HPH as described under Methods. **(A, B)** Levels of cytokines and chemokines were assessed by ELISA. N  =  4-5 rats for each group **(C, D)** Immunohistochemistry was performed with indicated antibodies as described under Methods and quantified by Image **J.** N  =  5 rats for each group.

## Supporting information

S1 File Image data underlying Fig 3C. CD3, CD45, and F480.
(ZIP)

S2 File Quantitative data underlying Fig 3A-B and D. (ZIP)

## References

[1] Yuan Z, Chen J, Chen D, Xu G, Xia M, Xu Y, et al. Megakaryocytic leukemia 1 (MKL1) regulates hypoxia induced pulmonary hypertension in rats. PLoS One. 2014;9(3):e83895. doi: 10.1371/journal.pone.0083895 24647044 PMC3960100

