# Supplementary material for: Megakaryocytic Leukemia 1 (MKL1) Regulates Hypoxia Induced Pulmonary Hypertension in Rats
Source: PLoS One. 2014 Mar 19;9(3):e83895. doi: 10.1371/journal.pone.0083895 (PMC3960100; doi:10.1371/journal.pone.0083895)
Supplement: Figure S1 — (A, B) Sprague Dawley rats were injected with lentiviral particles carrying shRNA targeting MKL1 or random shRNA (SCR) and induced to develop HPH as described under Methods. MKL1 mRNA (A) and protein (B) levels in aortic arteries were assessed by qPCR and immunohistochemistry. N = 5 mice for each group (C, D) A10 cells (C) and HPASMCs (D) were exposed to 1% O2 and harvested at indicated time points. mRNA and protein levels of MKL1 were measured by qPCR and Western. (PDF) [file pone.0083895.s001.pdf]

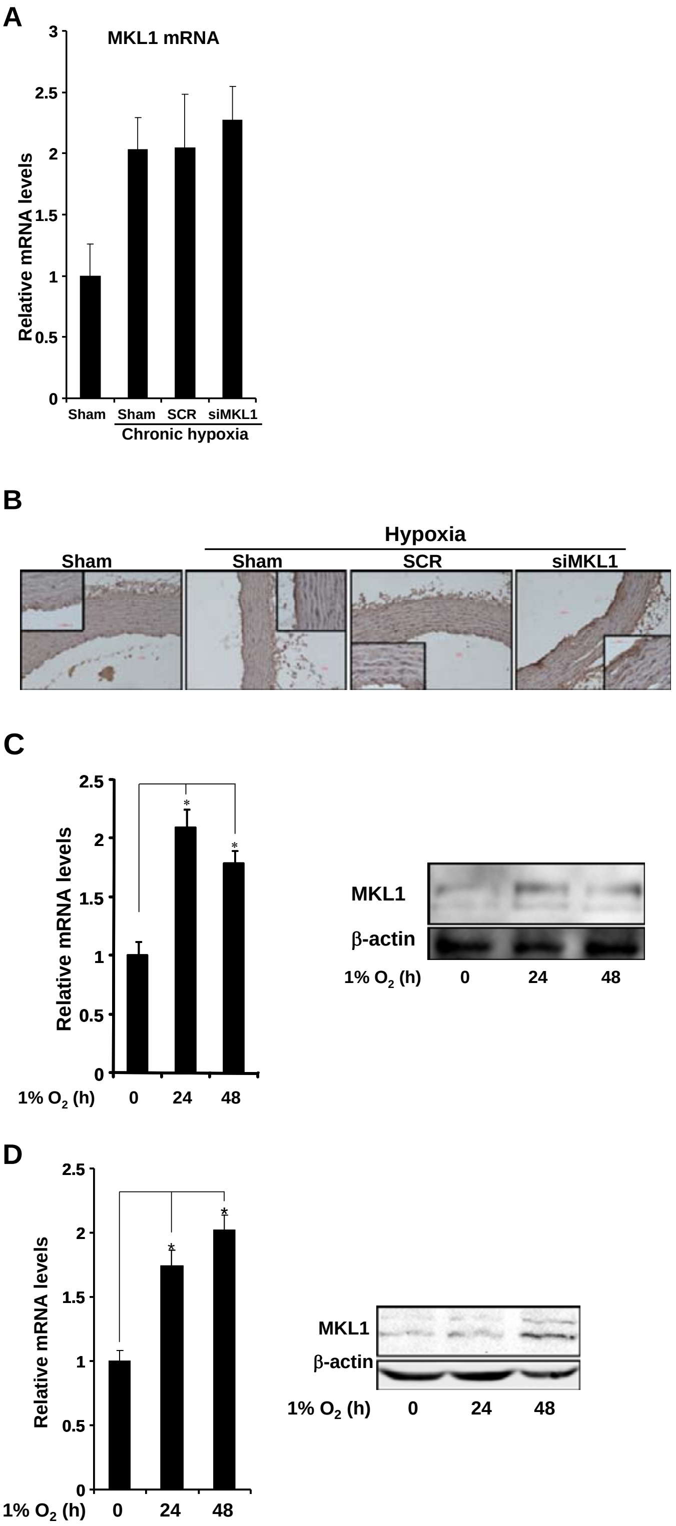

Figure S1: (**A, B**) Sprague Dawley rats were injected with lentiviral particles carrying shRNA targeting MKL1 or random shRNA (SCR) and induced to develop HPH as described under *Methods*. MKL1 mRNA (A) and protein (B) levels in aortic arteries were assessed by qPCR and immunohistochemistry. N=5 mice for each group (**C, D**) A10 cells (C) and HPASMCs (D) were exposed to 1% O_2 and harvested at indicated time points. mRNA and protein levels of MKL1 were measured by qPCR and Western.
