# Supplementary material for: Megakaryocytic Leukemia 1 (MKL1) Regulates Hypoxia Induced Pulmonary Hypertension in Rats
Source: PLoS One. 2014 Mar 19;9(3):e83895. doi: 10.1371/journal.pone.0083895 (PMC3960100; doi:10.1371/journal.pone.0083895)
Supplement: Figure S2 — (A, B) Sprague Dawley rats were injected with lentiviral particles carrying shRNA targeting MKL1 or random shRNA (SCR) and induced to develop HPH as described under Methods. Systemic blood pressure (A) and heart rate (B) were measured by using the PowerLab data acquisition system. N = 5 mice for each group (C–E) Sprague Dawley rats were injected with lentiviral particles carrying siMKL1 or SCR and maintained under normoxic conditions for 4 weeks. Pulmonary arterial pressure (C), systemic blood pressure (D), and heart rate (E) were recorded. N = 5 mice for each group (PDF) [file pone.0083895.s002.pdf]

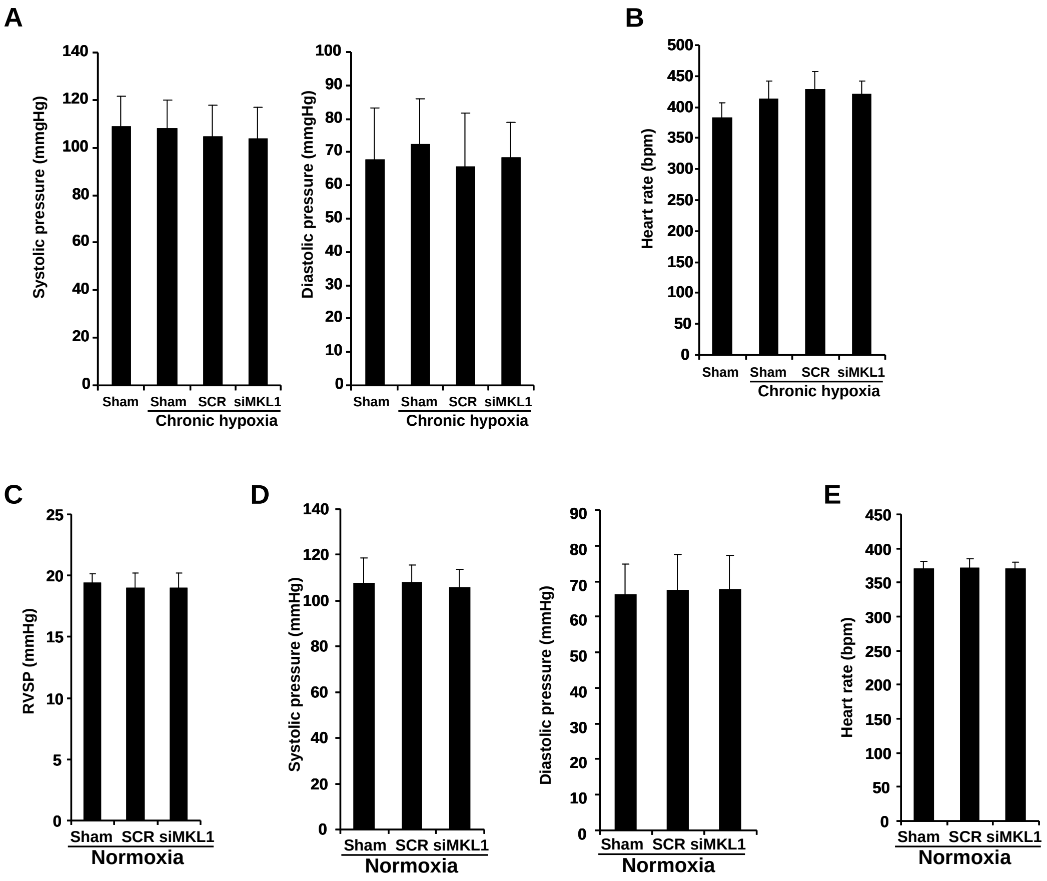

Figure S2: (A, B) Sprague Dawley rats were injected with lentiviral particles carrying shRNA targeting MKL1 or random shRNA (SCR) and induced to develop HPH as described under *Methods*. Systemic blood pressure (A) and heart rate (B) were measured by using the PowerLab data acquisition system. N=5 mice for each group (**C-E**) Sprague Dawley rats were injected with lentiviral particles carrying siMKL1 or SCR and maintained under normoxic conditions for 4 weeks. Pulmonary arterial pressure (C), systemic blood pressure (D), and heart rate (E) were recorded. N=5 mice for each group
