# Supplementary material for: Megakaryocytic Leukemia 1 (MKL1) Regulates Hypoxia Induced Pulmonary Hypertension in Rats
Source: PLoS One. 2014 Mar 19;9(3):e83895. doi: 10.1371/journal.pone.0083895 (PMC3960100; doi:10.1371/journal.pone.0083895)
Supplement: Table S1 — Real-time quantitative PCR primers. (PDF) [file pone.0083895.s003.pdf]

Table 1: Real-time quantitative PCR primers:

Gene	Species	Sequence	NCBI reference number	Amplicon size (bp)
fibronectin	rat	Forward: GACTCGCTTTGACTTCACCAC<br>Reverse: GCTGAGACCCAGGAGACCAC	NM_019143.2	150
TGF-β	rat	Forward: TAATGGTGGACCGCAACAACG<br>Reverse: GGCACTGCTTCCCGAATGTCT	NM_021578.2	100
Collagen type I α1	rat	Forward: TACAGCACGCTTGTGGATGG<br>Reverse: CAGATTGGGATGGAGGGAGTT	NM_053304.1	194
	human	Forward: TGCGATGACGTGATCTGTGA<br>Reverse: TTGGTCGGTGGGTGACTCTG	NM_000088.3	108
Collagen type I α2	rat	Forward: TGACCAGCCTCGCTCACAG<br>Reverse: CAATCCAGTAGTAATCGCTCTTCCA	NM_053356.1	185
	human	Forward: ATGCCTAGCAACATGCCAATC<br>Reverse: CAGCAAAGTTCCCACCGAGA	NM_000089.3	185
MKL1	rat	Forward: TCACCCACTCAGGTTCTTTCTCA<br>Reverse: GACTTCGGTTGGCTTTGCTT	XM_001077101.3	179
	human	Forward: GGCTTGAGGAACCATTTTCCT<br>Reverse: ACCCCCACTTCTCTGCTGAA	NM_020831.3	91
β-actin	rat	Forward: GACGTTGACATCCGTAAAGACC<br>Reverse: CTAGGAGCCAGGGCAGTAATCT	NM_031144.3	113
	human	Forward: CAGTCGGTTGGAGCGAGCAT<br>Reverse: GGACTTCCTGTAACAACGCATCTC	NM_001101.3	116
Collagen type III α1	rat	Forward: CCTCCCAGAACATTACATACCAC<br>Reverse: GACTGTCTTGCTCCATTCACCA	NM_032085.1	194
