# Supplementary figures and images for: Correction: Megakaryocytic Leukemia 1 (MKL1) Regulates Hypoxia Induced Pulmonary Hypertension in Rats
Source: PLoS One. 2026 May 27;21(5):e0350157. doi: 10.1371/journal.pone.0350157 (PMC13215492; doi:10.1371/journal.pone.0350157)

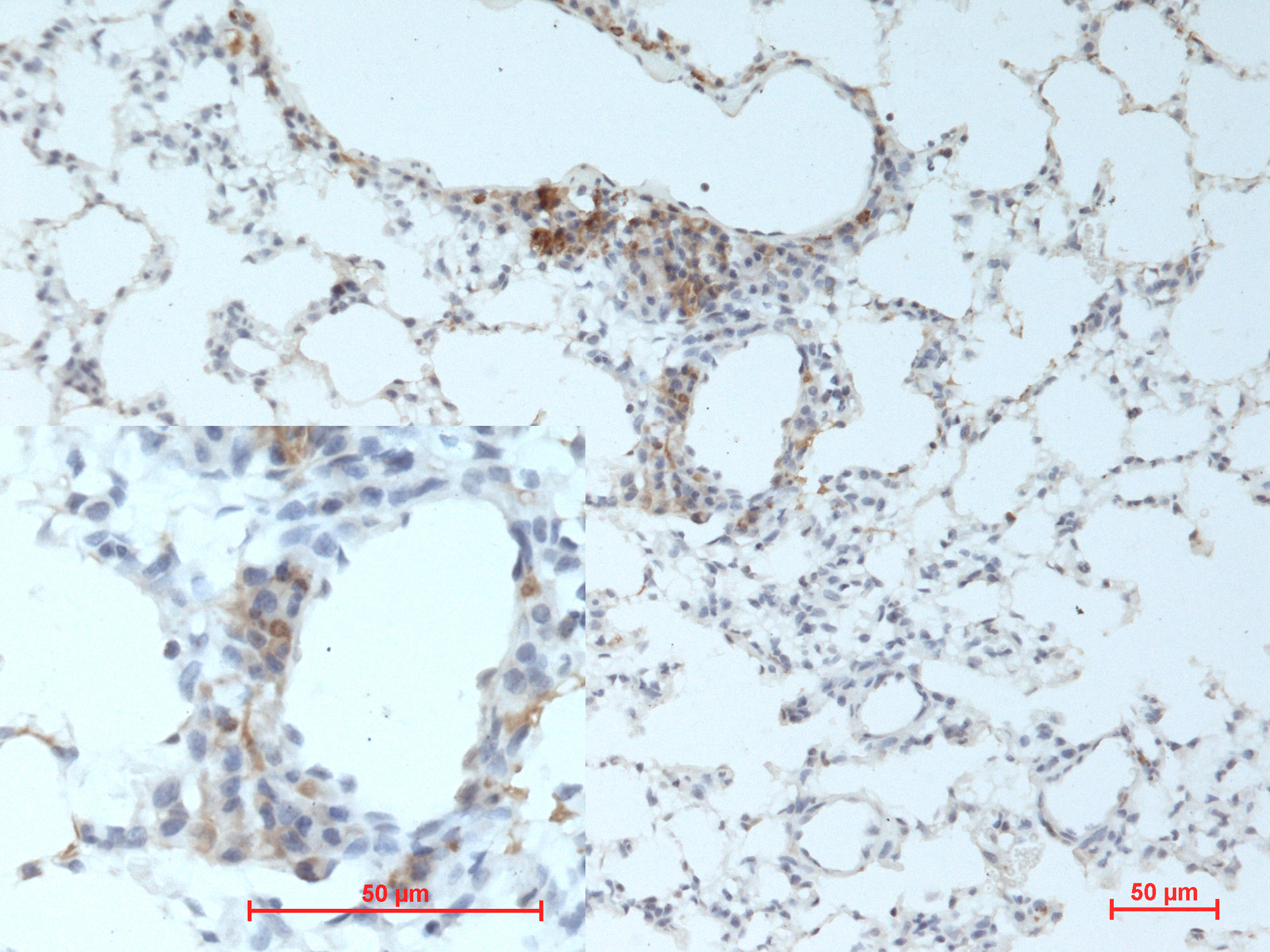

Supplement: S1 File — CD3, CD45, and F480. (ZIP) [file pone.0350157.s001.zip › S1 File/CD3/Hypoxia SCR.jpg]

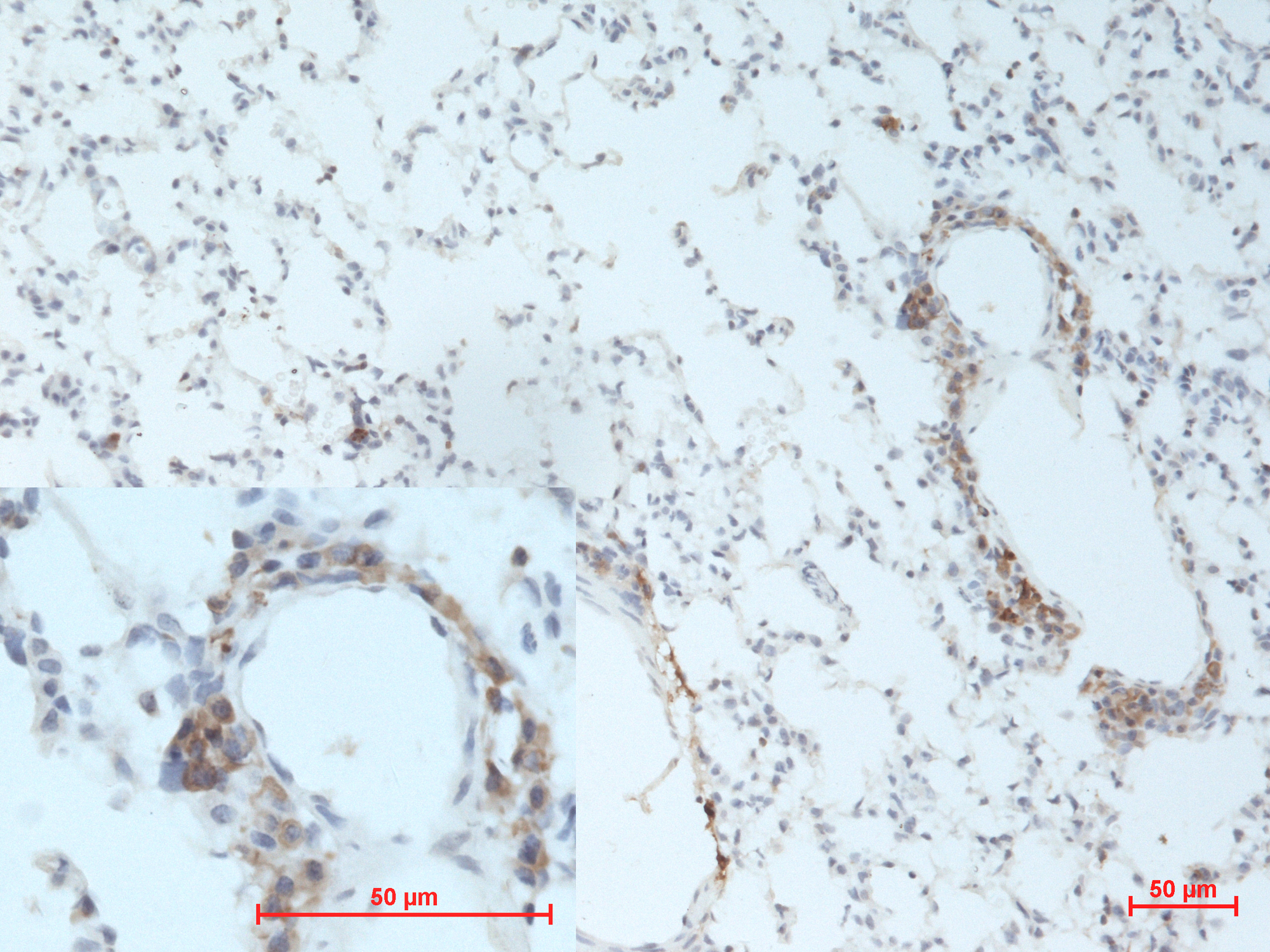

Supplement: S1 File — CD3, CD45, and F480. (ZIP) [file pone.0350157.s001.zip › S1 File/CD3/Hypoxia sham.jpg]

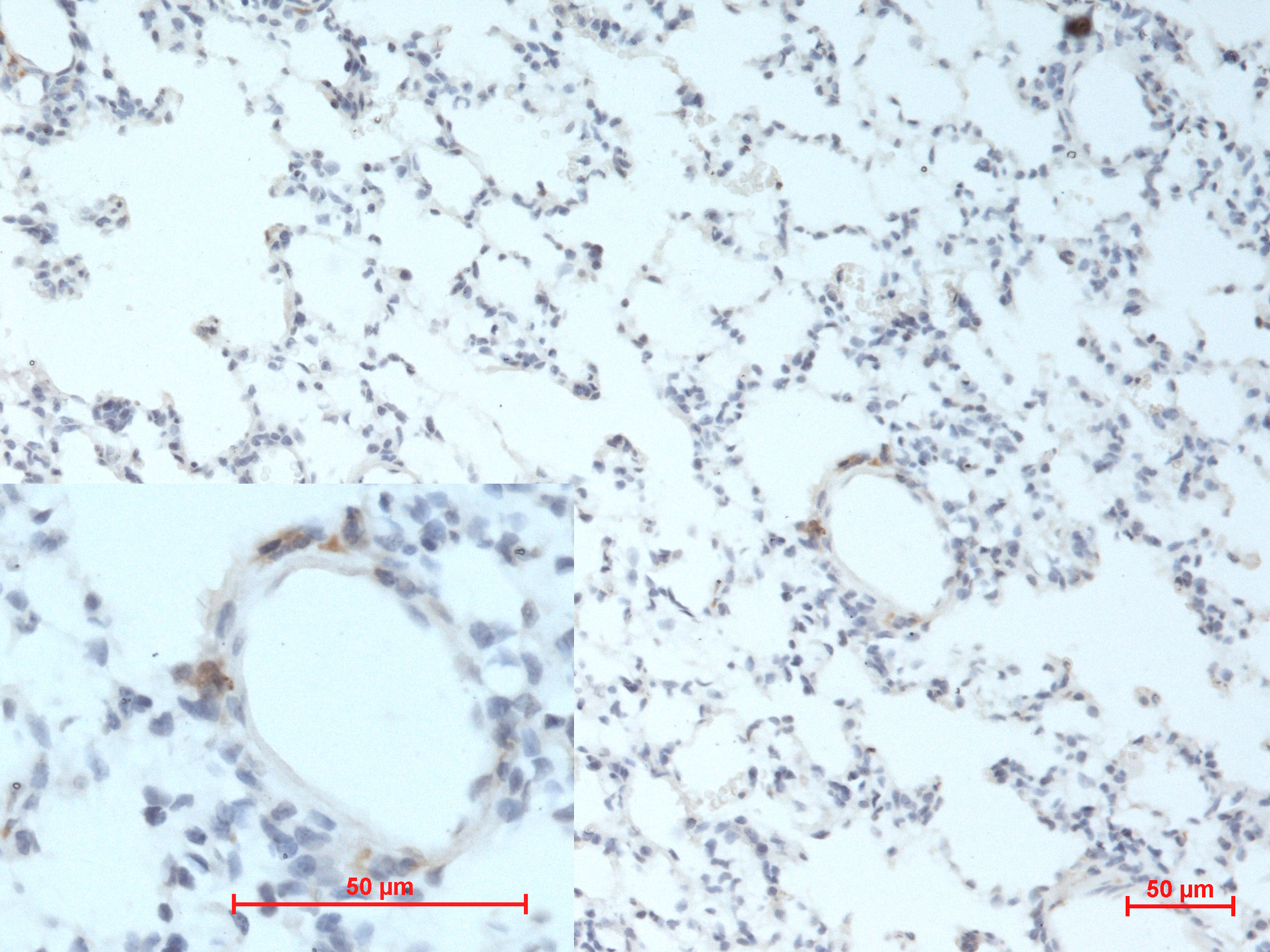

Supplement: S1 File — CD3, CD45, and F480. (ZIP) [file pone.0350157.s001.zip › S1 File/CD3/Hypoxia siMKL1.jpg]

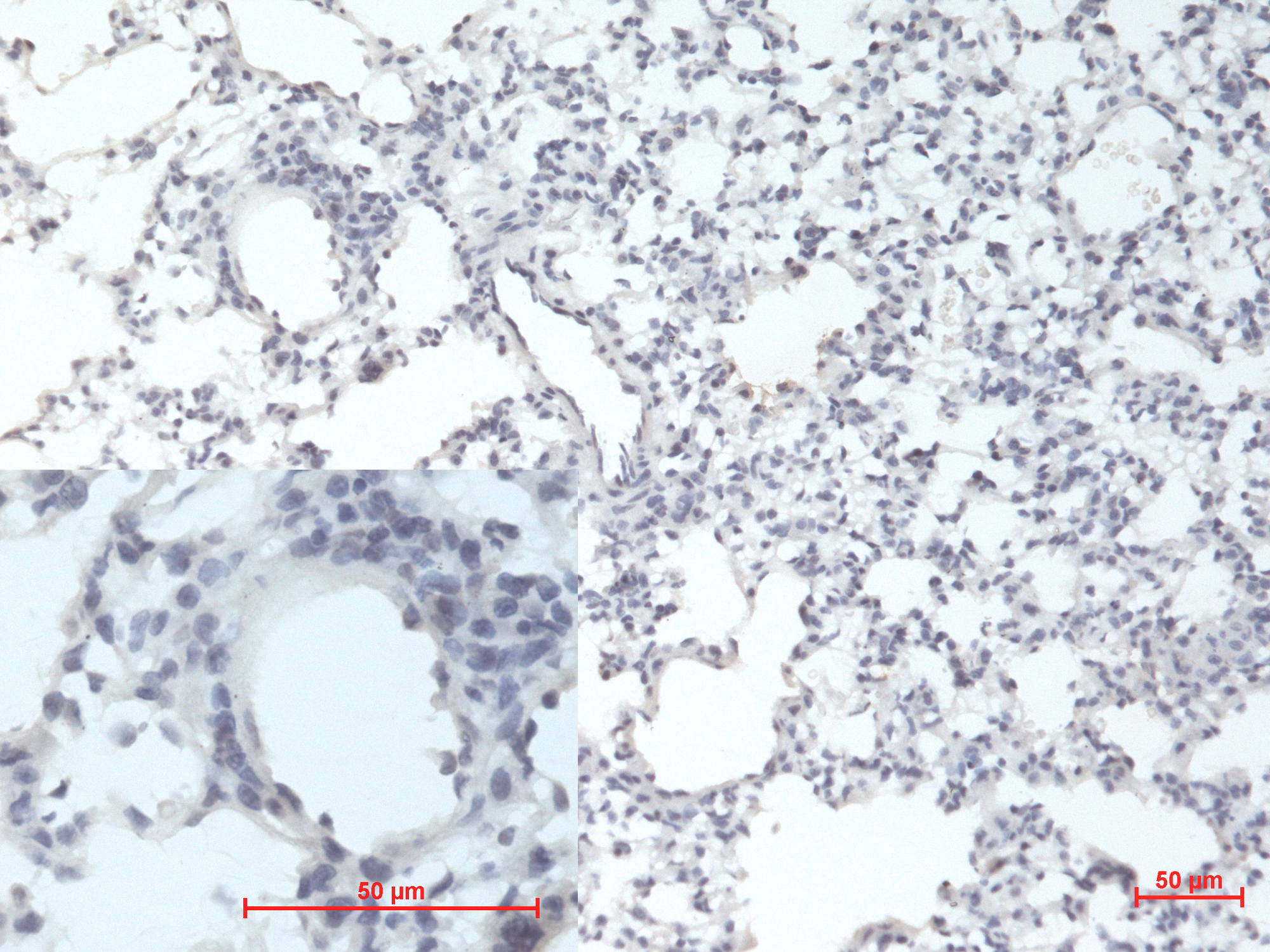

Supplement: S1 File — CD3, CD45, and F480. (ZIP) [file pone.0350157.s001.zip › S1 File/CD3/sham.jpg]

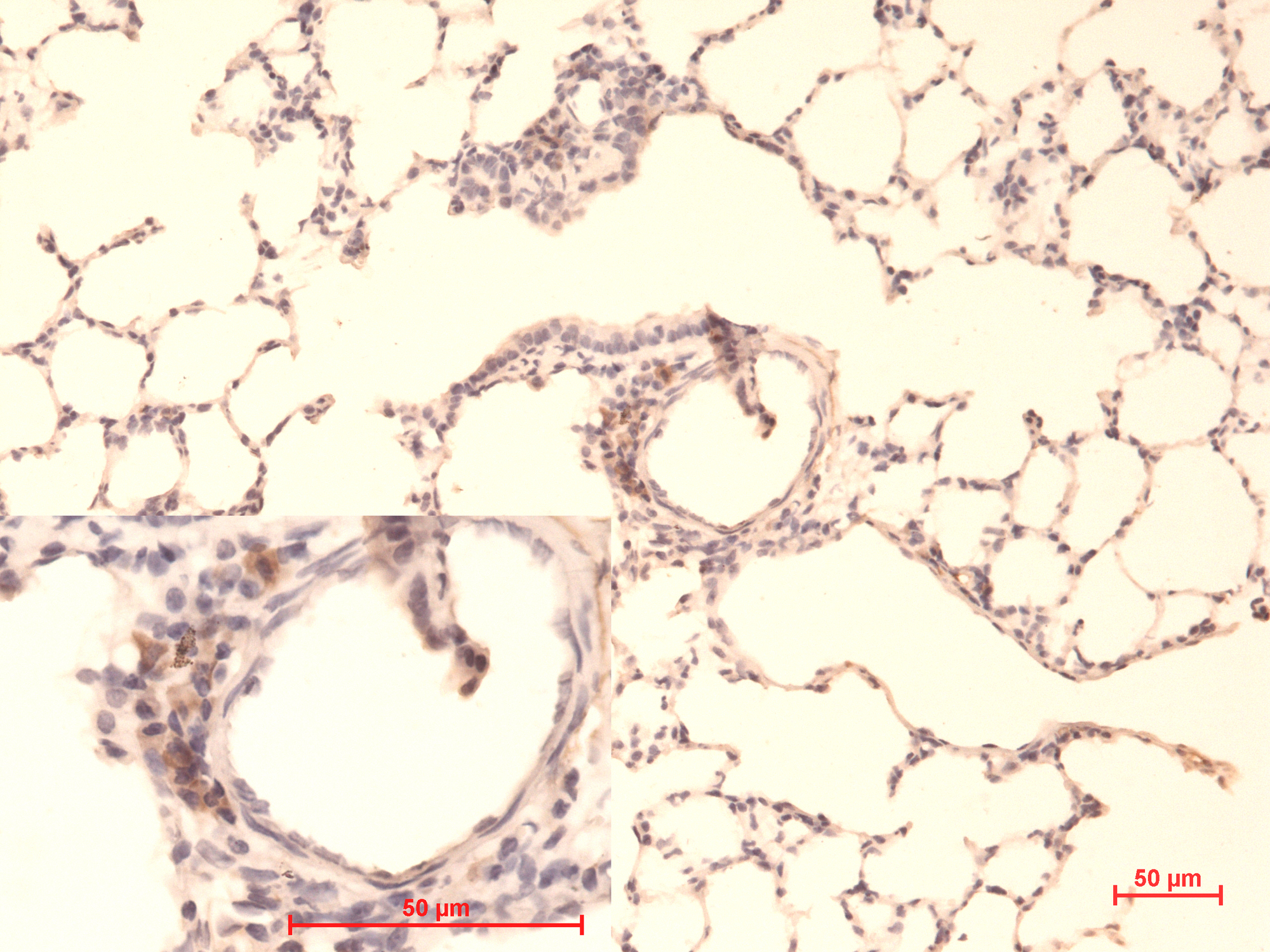

Supplement: S1 File — CD3, CD45, and F480. (ZIP) [file pone.0350157.s001.zip › S1 File/CD45/Hypoxia SCR.jpg]

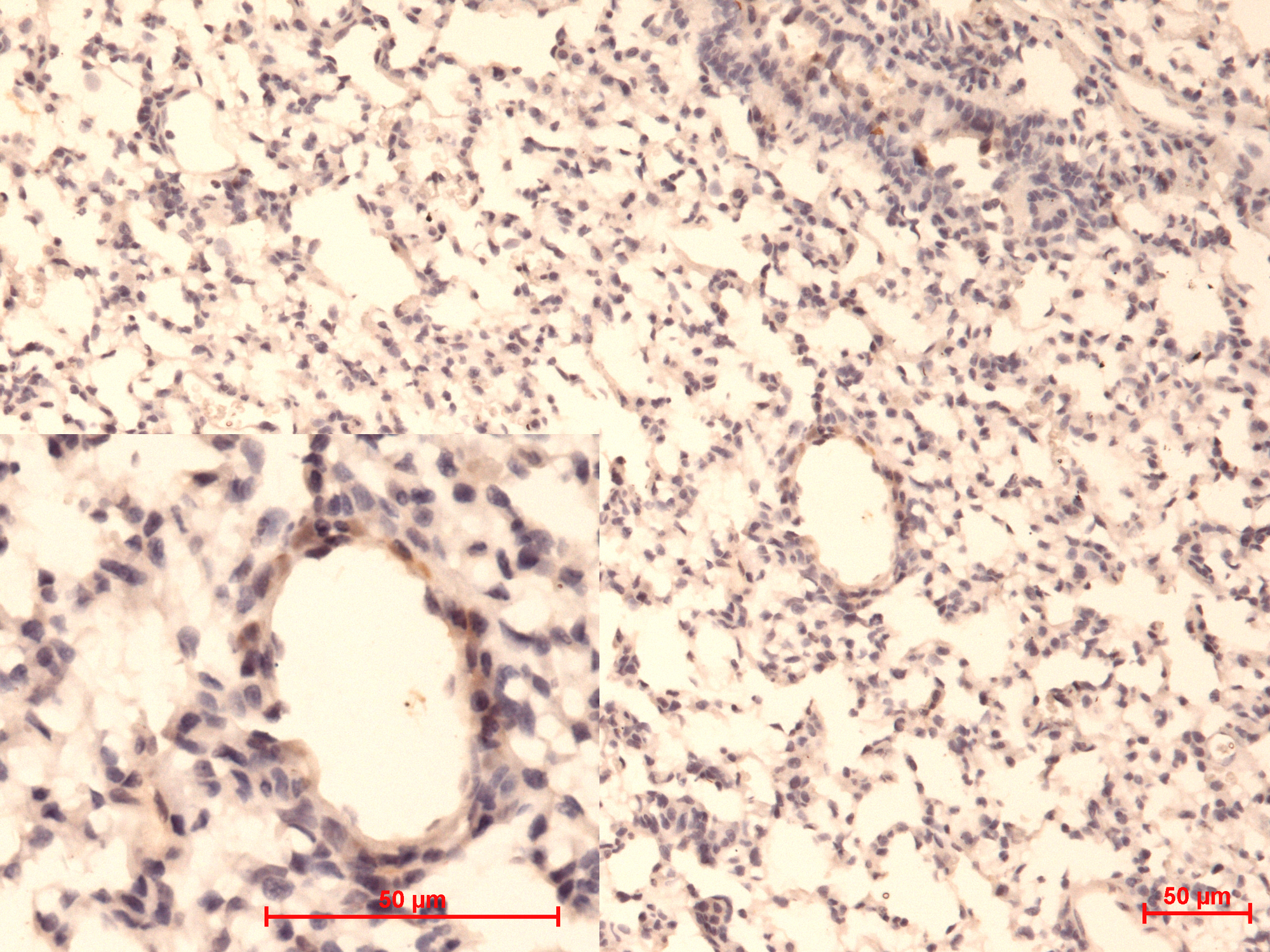

Supplement: S1 File — CD3, CD45, and F480. (ZIP) [file pone.0350157.s001.zip › S1 File/CD45/Hypoxia sham.jpg]

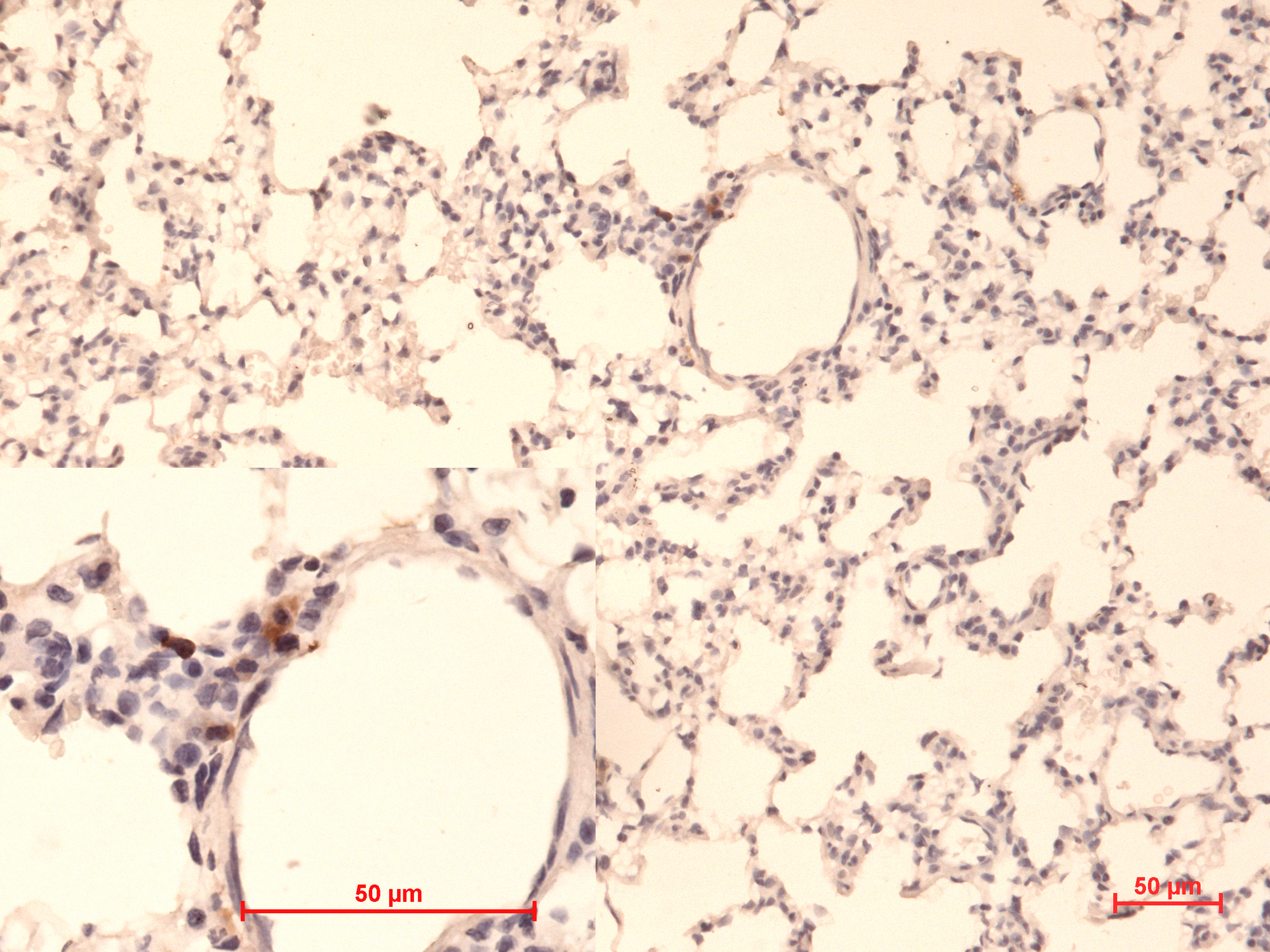

Supplement: S1 File — CD3, CD45, and F480. (ZIP) [file pone.0350157.s001.zip › S1 File/CD45/Hypoxia siMKL1.jpg]

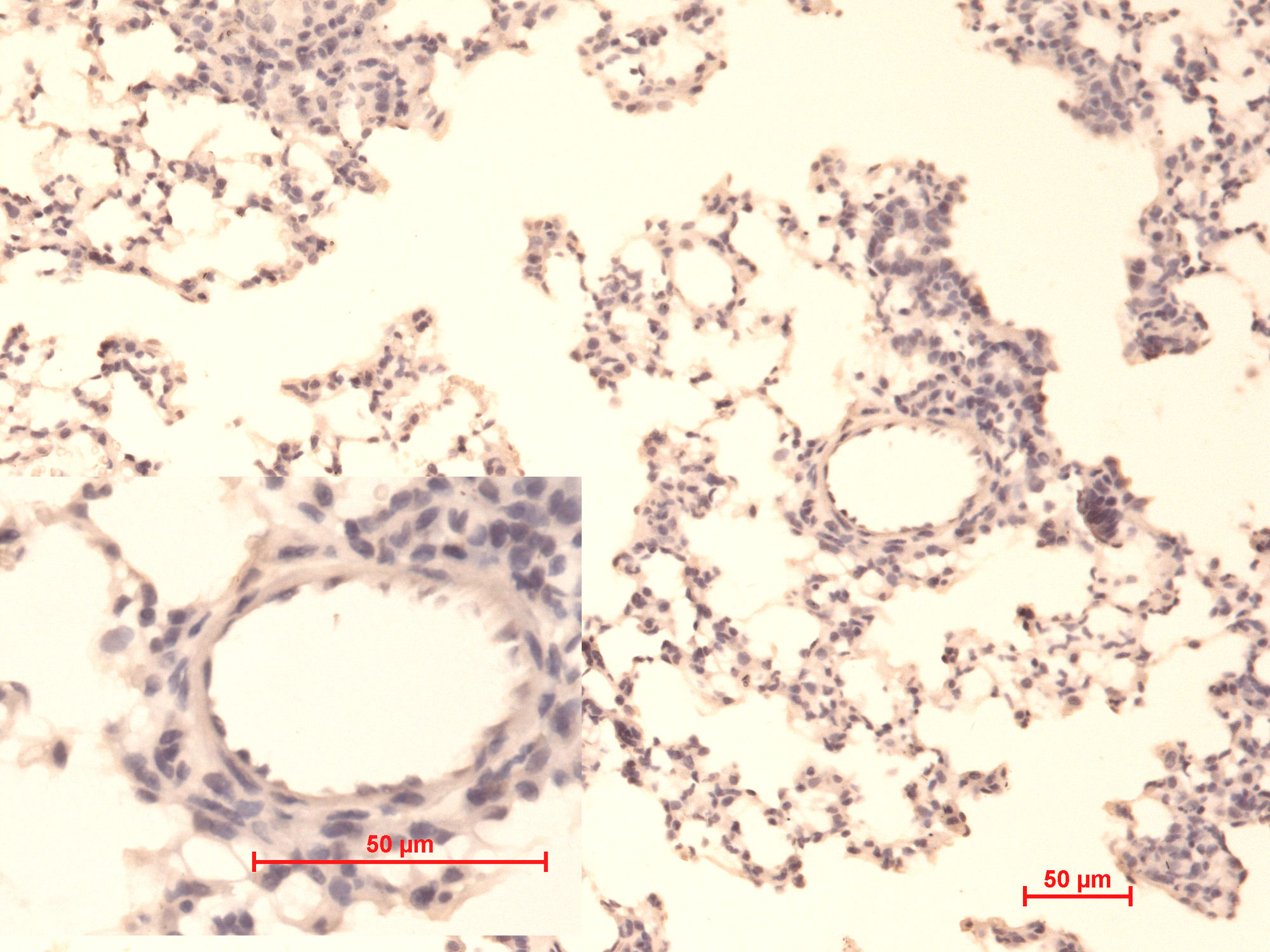

Supplement: S1 File — CD3, CD45, and F480. (ZIP) [file pone.0350157.s001.zip › S1 File/CD45/sham.jpg]

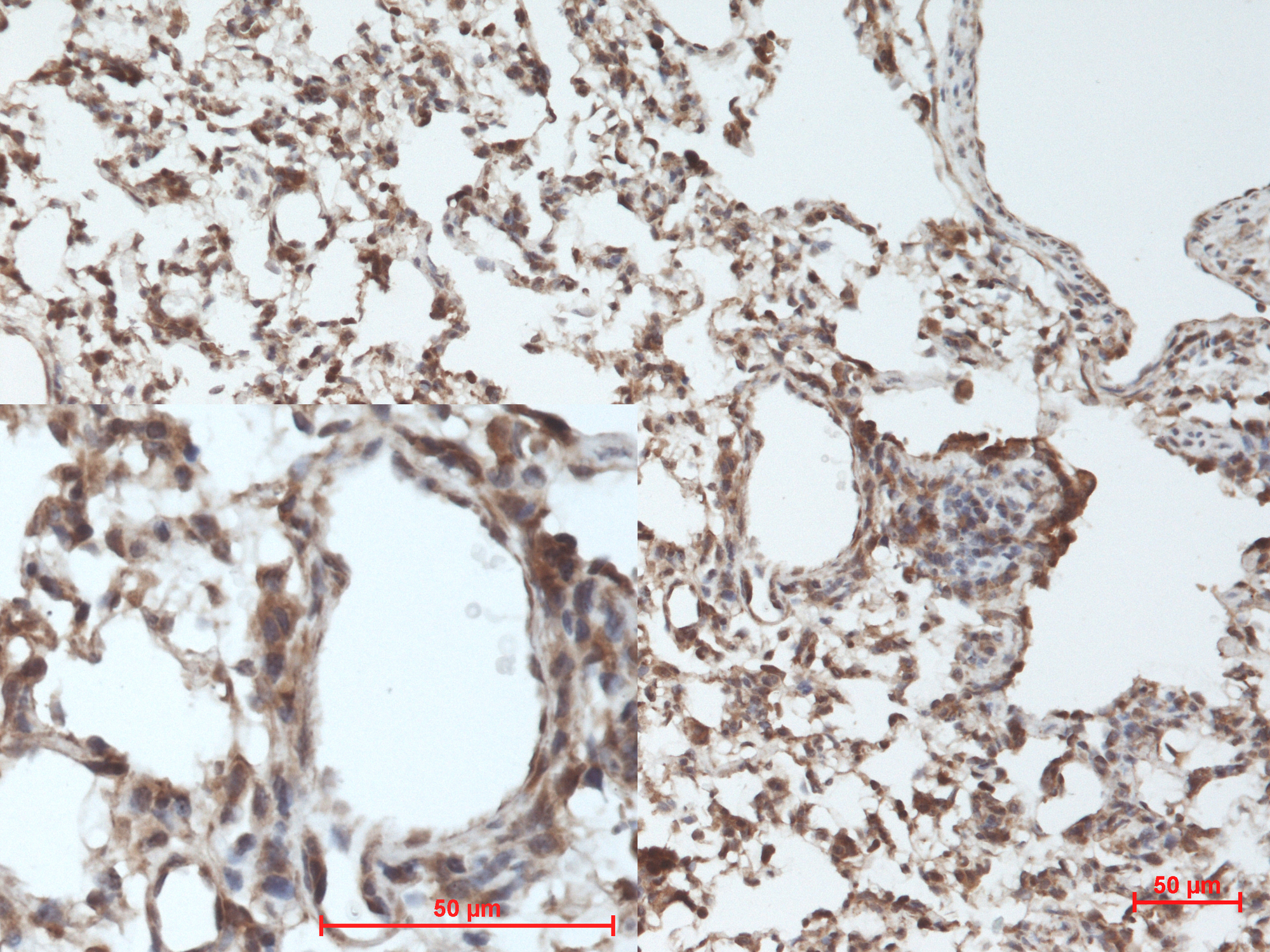

Supplement: S1 File — CD3, CD45, and F480. (ZIP) [file pone.0350157.s001.zip › S1 File/F480/Hypoxia SCR.jpg]

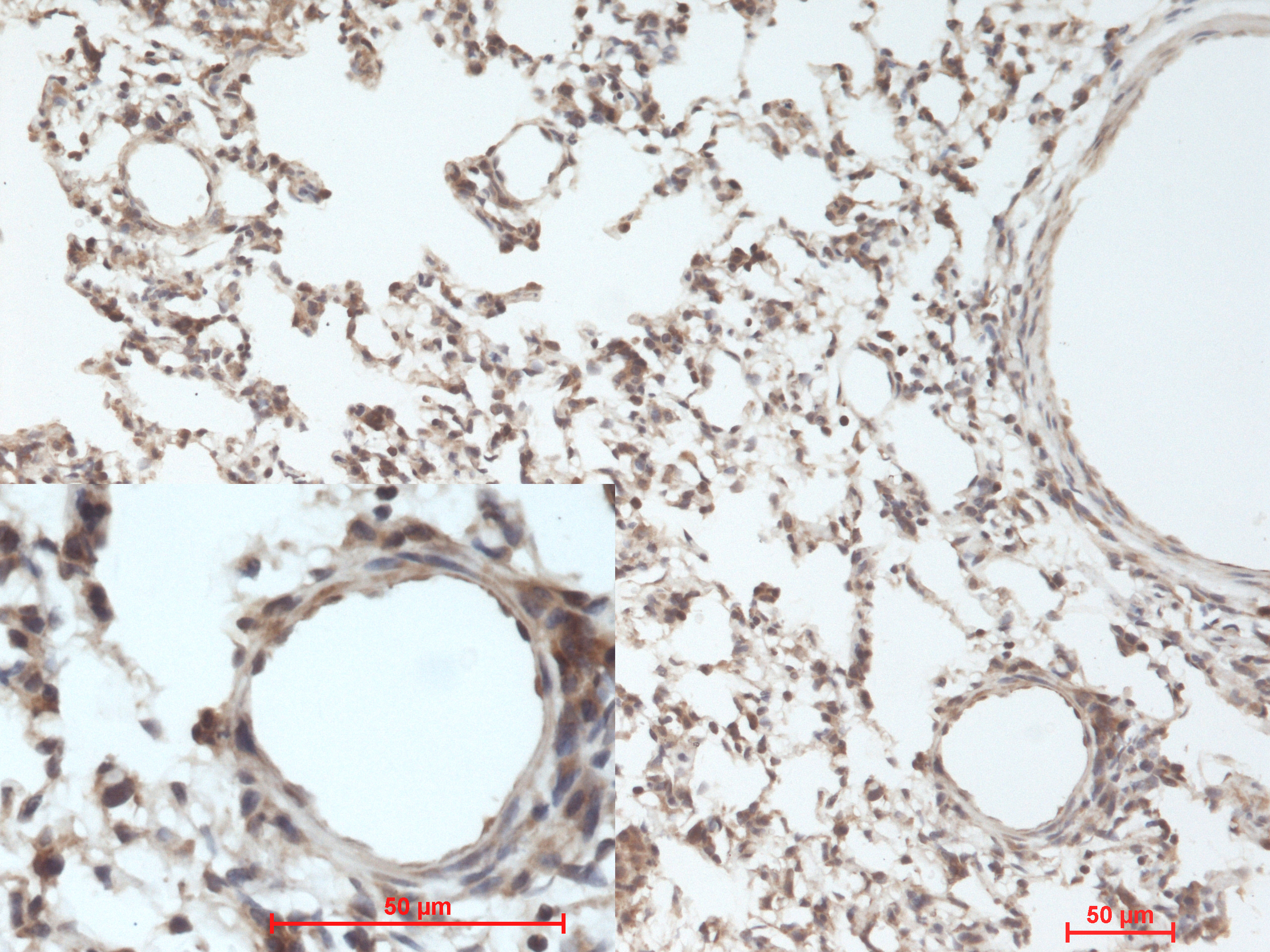

Supplement: S1 File — CD3, CD45, and F480. (ZIP) [file pone.0350157.s001.zip › S1 File/F480/Hypoxia sham.jpg]

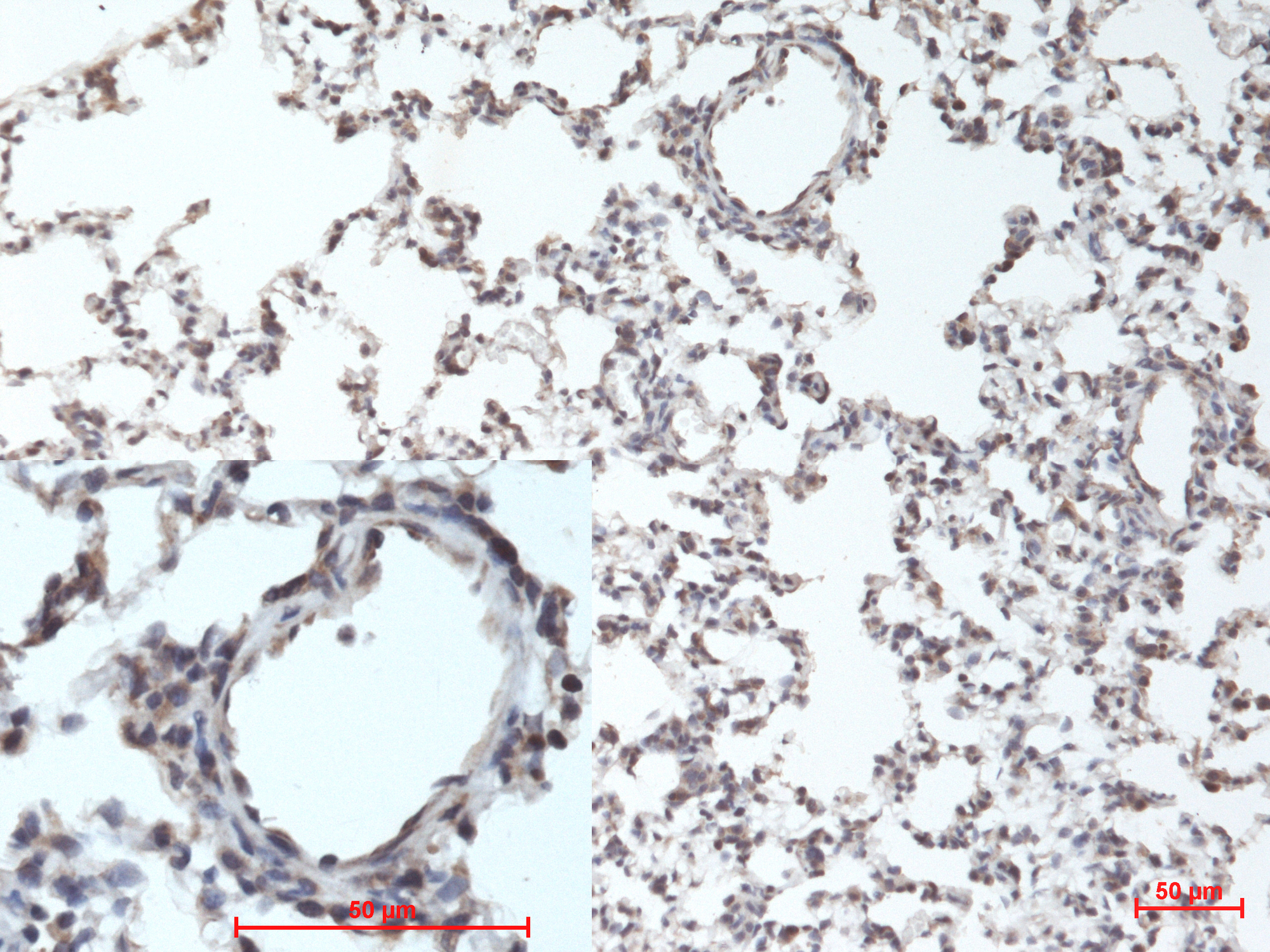

Supplement: S1 File — CD3, CD45, and F480. (ZIP) [file pone.0350157.s001.zip › S1 File/F480/Hypoxia siMKL1.jpg]

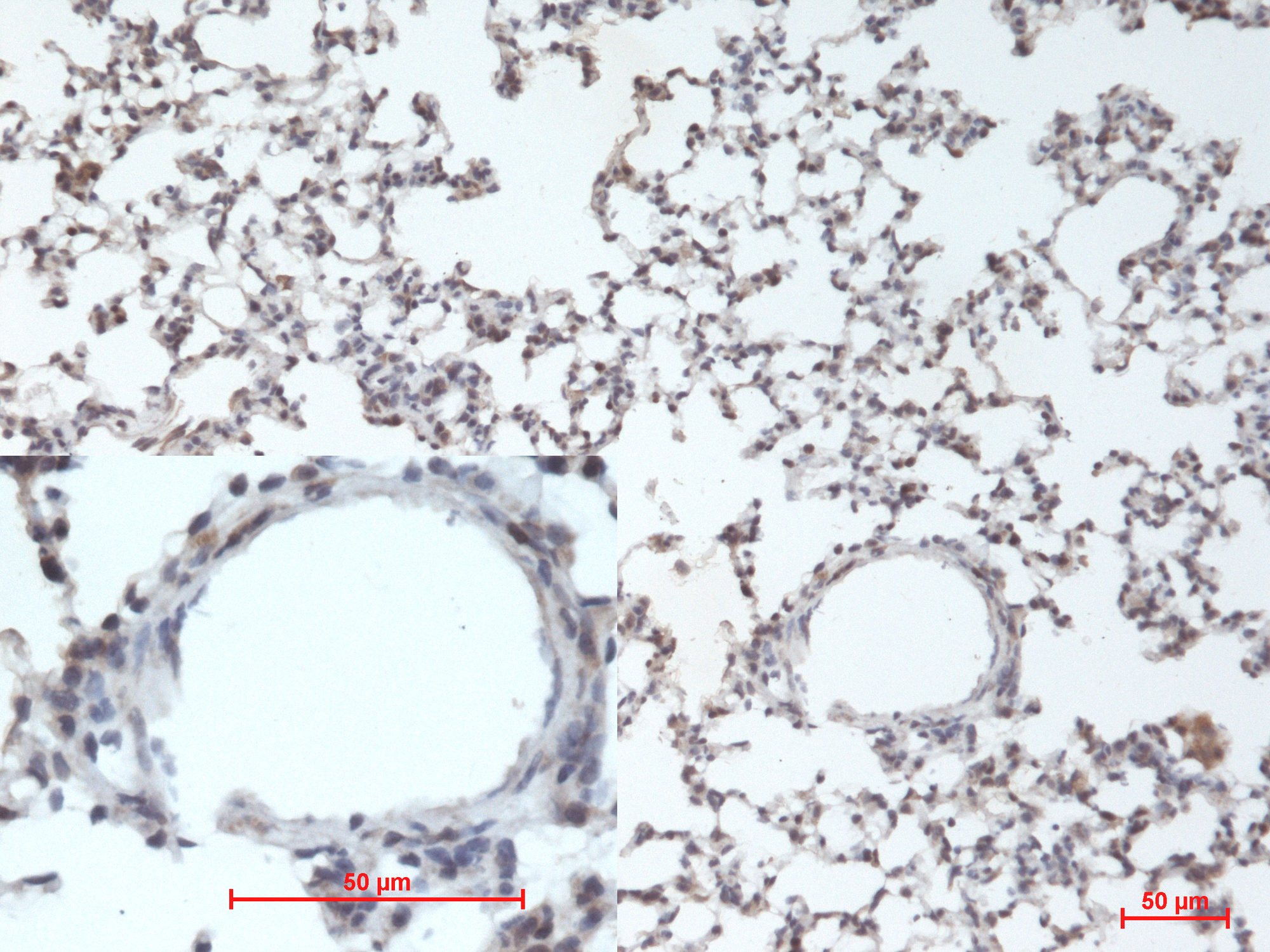

Supplement: S1 File — CD3, CD45, and F480. (ZIP) [file pone.0350157.s001.zip › S1 File/F480/sham.jpg]
